# County-level factors associated with a mismatch between opioid overdose mortality and availability of opioid treatment facilities

**DOI:** 10.1371/journal.pone.0301863

**Published:** 2024-04-05

**Authors:** John G. Rizk, Jannat Saini, Kyungha Kim, Uzma Pathan, Danya M. Qato

**Affiliations:** 1 Department of Practice, Sciences and Health Outcomes Research, University of Maryland School of Pharmacy, Baltimore, Maryland, United States of America; 2 Department of Epidemiology and Public Health, University of Maryland School of Medicine, Baltimore, Maryland, United States of America; University of New Mexico Health Sciences Center, UNITED STATES

## Abstract

**Background:**

Opioid overdose deaths in the United States remain a major public health crisis. Little is known about counties with high rates of opioid overdose mortality but low availability of opioid use disorder (OUD) treatment facilities. We sought to identify characteristics of United States (US) counties with high rates of opioid overdose mortality and low rates of opioid treatment facilities.

**Methods:**

Rates of overdose mortality from 3,130 US counties were compared with availability of opioid treatment facilities that prescribed or allowed medications for OUD (MOUD), from 2018-2019. The outcome variable, “risk-availability mismatch” county, was a binary indicator of a high rate (above national average) of opioid overdose mortality with a low (below national average) rate of opioid treatment facilities. Covariates of interest included county-level sociodemographics and rates of insurance, unemployment, educational attainment, poverty, urbanicity, opioid prescribing, depression, heart disease, Gini index, and Theil index. Multilevel logistic regression, accounting for the clustering of counties within states, was used to determine associations with being a “risk-availability mismatch” county.

**Results:**

Of 3,130 counties, 1,203 (38.4%) had high rates of opioid overdose mortality. A total of 1,098 counties (35.1%) lacked a publicly-available opioid treatment facility in 2019. In the adjusted model, counties with an additional 1% of: white residents (odds ratio, OR, 1.02; 95% CI, 1.01-1.03), unemployment (OR, 1.11; 95% CI, 1.05-1.19), and residents without insurance (OR, 1.04; 95% CI, 1.01-1.08) had increased odds of being a mismatch county. Counties that were metropolitan (versus non-metropolitan) had an increased odds of being a mismatch county (OR, 1.85; 95% CI, 1.45-2.38).

**Conclusion:**

Assessing mismatch between treatment availability and need provides useful information to characterize counties that require greater public health investment. Interventions to reduce overdose mortality are unlikely to be effective if they do not take into account diverse upstream factors, including sociodemographics, disease burden, and geographic context of communities.

## Introduction

Drug overdose deaths continue to constitute a major public health crisis. Opioids were involved in 75.4% (approximately 80,411) of these deaths in 2021 [1]. While historically, drug overdose deaths were concentrated in low-income, primarily white communities [2], more recent evidence suggests that the racial and ethnic profile of the United States (US) overdose crisis has changed in the past decade. In 2020 and 2021, the overdose death rate among non-Hispanic Black individuals was greater than those of non-Hispanic white individuals [3]. Additionally, beginning in 2016 and through 2020, a shift was seen in the urbanicity of the epidemic, with higher opioid overdose death rates in metropolitan areas compared to non-metropolitan areas [4,5]. Known risk factors for overdose mortality related to opioids at the individual level include clinical factors such as cardiovascular disease, comorbid mental disorders and psychological stress (e.g., depression), a history of substance use disorders, economic and community distress (e.g., low education, high unemployment), and characteristics such as male sex and middle age [6,7]. There is a paucity of evidence at the population level that has elucidated the relationship between population-level factors (e.g., unemployment, health insurance coverage) and areas where the availability does not meet the need for opioid use disorder (OUD) treatment.

Medications for OUD (MOUDs) are the standard and most effective treatment for OUD. Evidence from clinical trials has shown that the three MOUDs – buprenorphine, extended-release naltrexone, and methadone – are effective in reducing opioid use and all-cause mortality compared to not receiving an MOUD [8–12]. Despite the strong evidence base, access to MOUD is limited by low facility and provider uptake [13–16], economic and community factors, as well as persistent stigma surrounding OUD and MOUD [17]. Access to methadone for the treatment of OUD is especially challenging as it is restricted to licensed methadone maintenance treatment programs [18].

Buprenorphine, unlike methadone, is available via prescription and can be picked up at a community pharmacy. The recent Mainstreaming Addiction Treatment (MAT) Act of 2023 eliminated the X-waiver requirement that providers previously needed to prescribe buprenorphine. While eliminating the X-waiver was a step forward in expanding access to buprenorphine, particularly among underserved communities, concerns remain about the willingness of non-addiction medicine and non-psychiatry providers to expand their scope of practice [19] as well as the barriers to access that remain at the pharmacy level [20]. The use of naltrexone as a treatment for OUD has been limited by low adherence following induction [21]. This is due to the fact that naltrexone cannot be initiated without a week-long medically supervised withdrawal [22].

We focus our paper on opioid treatment facilities, since they are the most comprehensive setting for the provision of OUD treatment. These facilities integrate interdisciplinary care consisting of a combination of MOUD and counseling [23]. Specifically, facilities often offer a wide range of services including both methadone (opioid treatment program facilities) and buprenorphine, counseling, support groups, medical monitoring, and other ancillary services [24]. These facilities can thus provide more intensive support, particularly for individuals with complex medical or mental health needs.

We sought to identify regions with a mismatch between availability of facilities that either provided or allowed MOUDs and opioid overdose mortality rates. Since availability of facilities and opioid overdose mortality rates vary widely within every state, we sought to use county-level data to gain a more granular understanding of the characteristics of US counties with a mismatch of high overdose burden and low facility availability.

Our paper helps build our understanding of the interrelationship between overdose mortality, treatment availability, and county-level demographics and characteristics. Such information can be leveraged to support interventions that improve equity in OUD care in areas of the country that are most in need.

## Methods

### Study population and data sources

Data for 3,130 counties in 50 states and Washington D.C. were collected from several sources accessed through PolicyMap [25]. These include the American Community Survey (ACS), the Centers for Disease Control and Prevention (CDC), the United States Bureau of Labor Statistics (BLS), and the Substance Abuse and Mental Health Services Administration (SAMSHA). Counties with missing drug overdose mortality data were excluded (n=9). We analyzed characteristics associated with low availability of facilities and high rates of opioid overdose mortality using a cross-sectional analysis design that combined county-level data from January 1, 2017, to December 31, 2021, the most updated data available through PolicyMap. The study was deemed exempt from the University of Maryland Baltimore Institutional Review Board (IRB), given the use of publicly-available county-level data. This study followed the Strengthening the Reporting of Observational Studies in Epidemiology (STROBE) guidelines for cross-sectional studies [26].

### Opioid treatment facility data

The availability of facilities in 2019 was obtained via PolicyMap from the SAMHSA Behavioral Health Treatment Services Locator, a product of SAMHSA’s Center for Behavioral Health Statistics and Quality (CBHSQ) [27]. The Locator is compiled from responses to CBHSQ’s annual surveys of treatment facilities (the National Survey of Substance Abuse Treatment Services and the National Mental Health Services Survey). This constitutes a list of 11,111 facilities (from the 3,130 included counties) that indicated that they provided or allowed MOUD. Facilities that allow patients to be on MOUD may not have the authority or capability to prescribe MOUD prescriptions themselves, but may still accommodate and support patients who are on MOUD as part of their treatment plan from another provider [28]. Geocoding was performed using the “points with layer data” feature in PolicyMap, which provides the ability to find the geography and layer data that each address sits in [29]. We used 2020 US American Community Survey (ACS) estimates of county populations as the denominator in these calculations [30].

### Buprenorphine provider data

We included data of physicians certified to prescribe buprenorphine in 2019 from the SAMHSA Buprenorphine Treatment Physician Locator to examine whether the number of available buprenorphine physicians is correlated with the availability of facilities, and to examine the proportion of counties that have below-average rates for both treatment facilities and buprenorphine providers [31]. A total of 49,890 buprenorphine physicians were identified. Geocoding by the same method used for facilities was used for buprenorphine providers. The 2020 ACS estimates of county population were used as the denominator in these calculations.

### Opioid overdose deaths

County-level rates of drug overdose mortality were obtained from the National Vital Statistics System multiple cause of death files from the CDC [32]. Consistent with the CDC definition [32], we defined drug overdose deaths using the Tenth Revision (ICD-10) of the International Classification of Disease underlying-cause-of-death codes for drug poisonings (overdose): X40-44 (unintentional), X60-64 (suicide), X85 (homicide), and Y10–Y14 (undetermined intent).

### Identifying risk-availability mismatch counties

#### Low and high overdose death rates

We used the national average drug overdose mortality rate of 20.67 per 100,000 population from 2018 as a threshold to divide counties with high and low opioid overdose death rates. This national average was derived using the same national PolicyMap data from the CDC and SAMSHA.

#### Low and high facility availability

We first identified facilities that indicated they provided or allowed medications, then used the average national rate of 4.72 facilities per 100,000 population as a threshold to divide counties into high and low facility availability groups. Counties lacking facilities or those that have missing facility availability data were given a facility per 100,000 population value of 0.

#### Outcome of interest: Risk-availability mismatch county status

We then flagged “risk-availability mismatch” counties as counties with below-average rates of facilities and above-average rates of opioid overdose mortality. Specifically, counties were defined as risk-availability mismatch or non-mismatch. Mismatch counties are those with above average rates of overdose mortality and below average rates of facility availability, whereas non-mismatch counties are those that have: a) above average rates of overdose mortality and above average rates of facility availability; or b) below average rates of overdose mortality and below average rates of facility availability; or c) below average rates of overdose mortality and above average rates of facility availability. Choropleth maps were created to highlight geographic variation in risk-availability mismatch and non-mismatch counties in the U.S. [33]. The focus of our paper is on the mismatch between facility availability, rather than buprenorphine provider availability, because facilities provide the diversity of available resources for patients with OUD, including provision of both methadone and buprenorphine, and a range of other services [24].

### Covariates

The county-level risk indicators included in the analysis were sociodemographic characteristics, clinical characteristics, urbanicity, and opioid prescription rates. County-level demographic characteristics included estimated percent of: population per age category (<18, 18-64, ≥65 years), sex (percent male), race (percent white), people living in poverty, people with less than a high school diploma, and people who are uninsured. The Gini index, a measure of income inequality, was categorized into 2 levels based on the national average: ≥0.45 or <0.45. A Gini index of <0.45 represents lower income inequality, while a Gini index ≥0.45 represents higher income inequality. These variables were all derived from the ACS [34]. Theil index of racial segregation, an index ranging from 0 to 1, was calculated by PolicyMap using the U.S. Census Bureau’s 2010 Decennial Census estimates [35]; values approaching 0 suggest that sub-areas have a composition similar to the larger area (i.e., even distribution, less segregation) and values approaching 1 suggest that the racial and ethnic composition of sub-areas within a larger area deviates from the larger area (i.e., non-uniform distribution, more segregation). We categorized the Theil index into 2 levels based on PolicyMap’s definition: ≥0.4 or <0.4. County-level rates of heart disease and depression were drawn from CDC Population Level Analysis and Community Estimates (PLACES) [36]. We selected these chronic conditions because they are considered risk factors for opioid overdose mortality [6]. County-level rates of unemployment were obtained from the BLS [37]. County-level urbanicity was categorized as either metropolitan or non-metropolitan using National Center for Health Statistics Urban-Rural Classification Scheme for Counties which is based on the Office of Management and Budget’s February 2013 delineation of metropolitan statistical areas (MSA) and micropolitan statistical areas [38]. Opioid prescription rates per 100 persons in 2019 were obtained from the CDC’s prescription data, which comes from the QuintilesIMS Transactional Data Warehouse; this is based on a sample of approximately 59,000 retail (non-hospital) pharmacies that dispense roughly 88% of all retail prescriptions in the U.S. [39].

### Statistical analysis

Descriptive statistics contrasted mismatch counties with non-mismatch counties using 2-sample comparison tests based on county-level demographics, socioeconomics, percent of uninsured residents, clinical characteristics, urbanicity, and opioid prescription rates.

To test for multicollinearity between variables, we first examined the correlation matrix to see if any of the variables have a high correlation (set at 0.8 or higher) with any other variable. Then, we examined multicollinearity through the Variance Inflation Factor (>10 is suggestive of collinearity). Tetrachoric and polychoric correlations were computed for categorical variables. Multicollinearity between variables was not observed, and all covariates were eventually included in the model.

Univariate regression was performed between the variables of interest and the outcome (defined by whether the county is classified as mismatch or not). The outcome was then regressed against covariates described previously using generalized linear mixed models (random intercepts to account for nesting of counties in states) [40]. All independent variables specified above were included in the final model. We tested interactions between urbanicity and different clinical and socioeconomic factors to investigate how associations between the variables and outcome vary based on urbanicity. Analyses were conducted using SAS 9.4 (Cary, NC). We considered *P* < 0.05 to be statistically significant and used 2-sided tests.

### Sensitivity analyses

To ensure the robustness of our findings, we conducted two sensitivity analyses that incorporate buprenorphine provider availability into the definition of mismatch. In the first sensitivity analysis, we defined mismatch as a mismatch between high overdose rates and below average buprenorphine provider availability (i.e. below average national rate of 16.16 buprenorphine providers per 100,000 population) and facility availability. In the second sensitivity analysis, we defined mismatch based only on buprenorphine provider availability.

Additionally, in order to assess the robustness of findings related to the decision to use national averages as a threshold to create dichotomous outcome variable, we categorized counties into 3 mutually exclusive groups: mismatch, non-mismatch, and "mid-group". To be considered “mid-group”, a county was required to have an overdose mortality rate and a facility availability rate within 0.5 standard deviation of the mean [41–44]. Multilevel logistic regression was then conducted comparing the mid-group counties and the mismatch counties separately using non-mismatch counties as a reference group.

## Results

In 2019, there were a total of 11,111 facilities (from the 3,130 included counties) that indicated that they provided or allowed for MOUD consumption. Of these, 6,182 (55.6%) facilities provided MOUD and 4,929 (44.4%) did not provide MOUD but allowed patients to be on them if provided by an outside prescriber or facility. Of the 6,182 facilities providing MOUD, 737 (11.9%) were facilities that only provided naltrexone. The facilities were located in 2,032 of the 3,130 US counties or county equivalents (64.92%). A total of 863 of 1,968 non-metropolitan counties (43.85%) lacked any publicly-listed facility, whereas a total of 235 of 1,162 metropolitan counties (20.22%) lacked any publicly-listed facility. A total of 1,203 (38.43%) counties had high rates of overdose mortality, which includes 721 (36.64%) non-metropolitan counties and 482 (41.48%) metropolitan counties.

### Characteristics of risk-availability mismatch counties

**Table 1** provides a descriptive comparison between risk-availability mismatch counties and non-mismatch counties. We identified 671 mismatch counties and 2,459 non-mismatch counties. Mismatch counties had greater proportions of their population that were white, unemployed, with depression, with heart disease, and were more likely to be metropolitan than non-metropolitan. Mismatch counties also had higher rates of opioid prescriptions per 100-person population. In addition, mismatch counties were less likely to be racially segregated.

**Table 1:** Characteristics of United States counties (n=3,130) with a risk-availability mismatch between drug overdose mortality and opioid treatment facilities ^a^.

	Mean (SD) values			
Characteristics	All included counties	Non-mismatch Counties	Mismatch Counties	p-value^b^
N (%)	3130	2459 (78.56)	671 (21.44)	N/A
Average rate of drug overdose deaths per 100,000	20.67 (8.54)	18.77 (7.86)	27.62 (7.27)	<0.001
Average number of facilities per 100,000	4.72 (7.37)	5.54 (8.08)	1.73 (1.70)	<0.001
Rate of opioid prescriptions per 100 population^c^	41.19 (33)	40.52 (32.23)	43.63 (35.59)	0.04
Sex, % male	50.36 (2.43)	50.41 (2.44)	50.17 (2.43)	0.02
Race, % white	76.83 (17.86)	76.47 (18.32)	78.17 (15.99)	0.01
Age, %				
<18	22.25 (3.60)	22.44 (3.58)	21.56 (3.57)	<0.01
18-64	58.54 (4.03)	58.39 (4.04)	59.09 (3.96)	<0.01
≥65 y	19.21 (4.72)	19.17 (4.68)	19.35 (4.86)	0.39
Theil Index, N (%)				
<0.4	1572 (50.22)	1171 (47.62)	401 (59.76)	<0.01
≥0.4	1558 (49.78)	1288 (52.38)	270 (40.24)	
Urbanicity, N (%)				
Metropolitan	1162 (37.12)	827 (33.63)	335 (49.93)	<0.01
Non-metropolitan	1968 (62.88)	1632 (66.37)	336 (50.07)	
Socioeconomic				
Poverty, %	14.42 (6.08)	14.43 (6.37)	14.36 (4.91)	0.77
Unemployment, %^d^	6.73 (2.26)	6.57 (2.24)	7.32 (2.21)	<0.01
Less than high school degree, %	12.06 (5.99)	11.98 (6.23)	12.35 (4.99)	0.11
Uninsured, %	9.65 (5.10)	9.65 (5.22)	9.63 (4.64)	0.91
Gini Index, N (%)				
<0.45	1639 (52.36)	1296 (52.70)	343 (51.12)	0.47
≥0.45	1491 (47.64)	1163 (47.30)	328 (48.88)	
Clinical Characteristics				
Heart disease, %	8.21 (1.57)	8.16 (1.56)	8.37 (1.60)	<0.01
Depression, %	21.10 (3.15)	20.82 (3.10)	22.16 (3.10)	<0.01

a. Mismatch counties are those with rates of drug overdose mortality above the national average for all counties (average=20.66 deaths per 100,000 population), and with rates below the national average in availability of OUD treatment facilities that that provide or allow medications (average=4.72 facilities per 100,000 population). Missing values for overdose mortality were identified for 9 counties, and were not included in the analysis.

b. P-values for numerical variables were derived using the independent sample t tests. P-values for categorical variables were derived using Pearson chi-square 2-way tests for independent samples.

c. Number of retail opioid prescriptions dispensed per 100 persons in 2019. Opioids include codeine phosphate, fentanyl citrate, hydrocodone bitartrate, hydromorphone hydrochloride, methadone hydrochloride, morphine sulfate, oxycodone hydrochloride, oxymorphone hydrochloride, propoxyphene hydrochloride, tapentadol hydrochloride, and tramadol hydrochloride, identified using the National Drug Code. Cough and cold formulations containing opioids, buprenorphine products typically used to treat opioid use disorder, and methadone dispensed through methadone maintenance treatment programs are excluded. Missing 46 counties (9 mismatch counties, 37 non-mismatch counties).

d. Missing 1 mismatch county.

### Adjusted characteristics of risk-availability mismatch counties

**Table 2** includes the multilevel logistic regression results for characteristics associated with risk-availability mismatch counties, adjusted for county-level demographics, urbanicity, opioid prescribing, racial segregation, socioeconomic factors, and clinical factors. Several factors were associated with higher odds of being a mismatch county, namely, higher percentage of residents who are white, uninsured, and unemployed. Counties with an additional 1% of white population (OR, 1.02; 95% CI, 1.01-1.03) and those with an additional 1% of residents without insurance (OR, 1.04; 95% CI, 1.01-1.08) had increased odds of being a mismatch county. A 1% increase in unemployment was associated with an increased odds (OR, 1.11; 95% CI, 1.05-1.19) of a county being a mismatch county. Counties that were metropolitan (versus non-metropolitan) also had an increased odds of being a mismatch county (OR, 1.85; 95% CI, 1.45-2.38). Clinical factors including percent of residents with heart disease (OR, 1.13; 95% CI, 0.97-1.32) and depression (OR, 1.04; 95% CI, 0.95-1.14) were not significantly associated with the outcome in the adjusted analysis, although depression rates were significantly associated with mismatch counties in the univariate regression (OR, 1.08; 95% CI, 1.02-1.15). Results for the unadjusted regression are available in S1 Table.

**Table 2:** United States county-level characteristics associated with a mismatch between opioid overdose mortality and availability of opioid treatment facilities, multivariable analysis (n=3,130).

Characteristics	Adjusted Odds Ratio (95% CI)
Rate of opioid prescriptions per 100 population	1.00 (1.00-1.00)
Sex, % male	0.96 (0.91-1.02)
Race, % white	1.02 (1.01-1.03)***
% Age 18-64 y	1.04 (0.99-1.10)
Theil Index	
<0.4	**1 (reference)**
≥0.4	1.04 (0.81-1.32)
Urbanicity	
Non-metropolitan	**1 (reference)**
Metropolitan	1.85 (1.45-2.38)***
**Socioeconomic factors**	
% Unemployment	1.11 (1.05-1.19)***
% Less than high school degree	1.02 (0.99-1.05)
% Poverty	0.95 (0.92-0.99)**
% Uninsured	1.04 (1.01-1.08)*
Gini Index	
<0.45	**1 (reference)**
≥0.45	0.92 (0.73-1.16)
**Clinical factors**	
% Heart disease	1.13 (0.97-1.32)
% Depression	1.04 (0.95-1.14)

*P<0.05

**P<0.01

***P<0.001. Model was adjusted for all variables above. To account for nesting of counties in states, a random effect model was used.

### Interactions

We identified significant interactions between urbanicity and unemployment rates, percent of uninsured residents, and depression rates (**Table 3**). In metropolitan counties, higher unemployment and depression rates were associated with 31% and 12% higher odds of being a mismatch county compared to a non-mismatch county, respectively. These significant associations were not observed for non-metropolitan counties. In non-metropolitan counties, a higher percentage of uninsured individuals was associated with 6% higher odds of a county being a mismatch county compared to a non-mismatch county. This significant association was not observed for metropolitan counties.

**Table 3:** Adjusted odds ratios of United States counties with a risk-availability mismatch, interaction for urbanicity with rates of unemployment, uninsured residents, and depression (n=3,130).

Characteristics	Adjusted Odds Ratio (95% CI)	
	Metropolitan (n=1,162)	Non-Metropolitan (n=1,968)
% Unemployment	1.31 (1.18-1.46)***	1.02 (0.94-1.11)
% Uninsured	1.01 (0.94-1.08)	1.06 (1.02-1.10)**
% Depression	1.12 (1.00-1.26)*	1.11 (0.97-1.26)

*P<0.05

**P<0.01

***P<0.001.

Model was adjusted for all variables above. To account for nesting of counties in states, a random effect model was used.

### Geographical distribution of risk-availability mismatch counties

**Fig 1:**
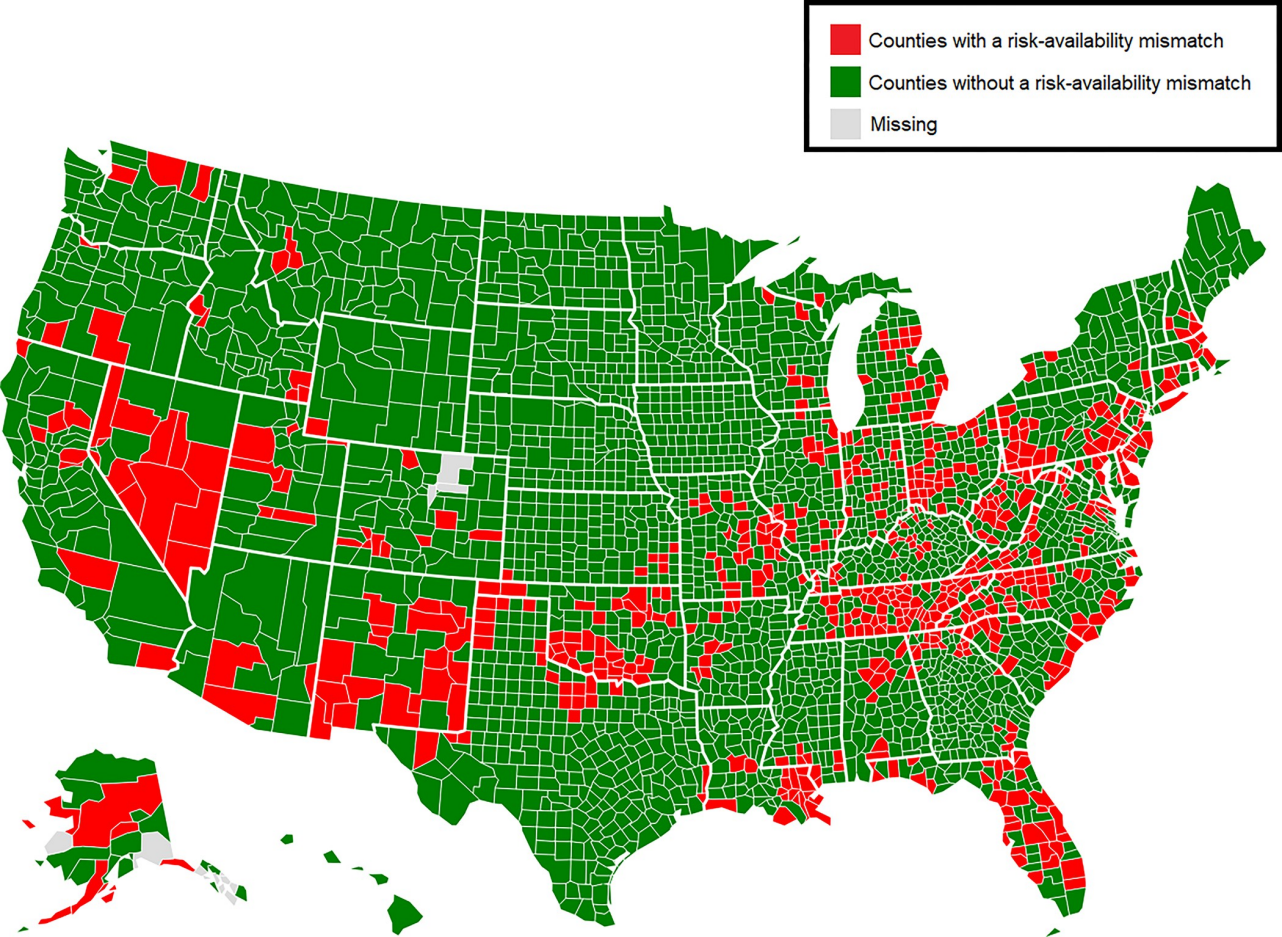
The geographic distribution of risk-availability mismatch counties. Mismatch counties are those with below national rates of opioid treatment facilities in 2019 and above national rates of overdose mortality in 2018.

Fig 1 shows that mismatch counties were concentrated throughout the states of New Mexico, Florida, Nevada, and Central Appalachia, eastern division of Missouri and the Metro East region of Illinois, and parts of New England (central Connecticut, eastern Massachusetts, and southern New Hampshire). Mismatch counties were also prevalent in Pennsylvania, Oklahoma, Ohio, Indiana, Louisiana, and Michigan. Non-mismatch counties were concentrated in Texas, Vermont, Maine, New York, Hawaii, Idaho, the Great Plains, and were also prevalent in Southern states (Alabama, Georgia, and Mississippi). While there are pockets of mismatch counties in the West coast, most counties there were non-mismatch.

In both sensitivity analyses where buprenorphine providers were taken into account, the multilevel logistic regression results were generally similar (with a few exceptions, for example insurance was no longer significant in the analysis that defined county mismatch status based on both buprenorphine provider and facility availability) to our primary analysis (S2 Table, S1 and S2 Figs). Additionally, sensitivity analysis that included mid-group counties yielded similar results to our primary analysis, where a higher percent of unemployment rates, uninsured residents, white residents, and a county being metropolitan (versus non-metropolitan) were associated with increased odds of being a mismatch county (S3 Table, S3 Fig).

## Discussion

We analyzed characteristics of counties that exhibited both high rates of overdose mortality and low facility availability. Risk-availability mismatch counties represent areas where the availability does not meet the need for MOUD treatment services. Our findings indicate that counties with higher rates of overdose mortality and who face greater challenges in accessing treatment also have high rates of clinical and social risk factors. This work underscores and adds greater granularity to previous research that has highlighted the potential benefit that increasing the delivery of MOUD through facilities, primary care providers (particularly after the elimination of the X-waiver), and addiction and psychiatry specialists in counties exhibiting a higher level of risk factors can result in a meaningful decrease in overdose deaths [8]. In terms of variation in facility-provision of MOUD across the US, we found higher rates of facility availability in metropolitan versus non-metropolitan counties. Overall, our results can help policymakers and advocates better understand county-level characteristics associated with care needs and resource availability so that levers to improve treatment access may be more effectively deployed. Our work also illuminates the multitude of population-level factors that drive access to OUD care.

Risk-availability mismatch counties had higher rates of residents who were white, uninsured, unemployed, and were more likely to be metropolitan. Economic deprivation is a risk factor for opioid overdoses in the US and independently contributes to declining life expectancy. This effect is exacerbated when access to resources is challenging [45]. It has been previously reported that a rise in unemployment also increases the risk of opioid-related mortality [46]. Manual labor occupations with higher injury risk are usually the most available employment opportunities in disadvantaged and economically precarious communities [47]. These injuries lead to chronic painful conditions that necessitate the use of opioids, thereby increasing the risk of overdose deaths [48]. Thus, OUD, and subsequent overdose death, often occurs amid economic and health problems that can lead to loss of insurance [49]. Such a relationship points to the need to not only ensure judicious use of opioids, but also the need to ensure occupational safety and expand access to health insurance and healthcare for patient populations that are at risk due to their economic condition [48].

The relationship between county socioeconomic features and depression, and a county being mismatch, varied by area urbanicity (Table 3). In metropolitan counties, increased unemployment and depression were associated with higher odds of a county being mismatch, but a significant association was not observed in non-metropolitan counties. While both overdose rates and facility availability are higher in metropolitan counties compared with non-metropolitan counties, the association we observed between unemployment rates and a county being mismatch in metropolitan counties may be explained by the *availability-proneness theory*. Non-prescription opioids, such as heroin, are more available in metropolitan compared to non-metropolitan areas [50]. The availability-proneness theory of drug use posits that drug use occurs when individuals who are prone (specifically vis-à-vis socioeconomic stressors such as unemployment and psychological stressors such as depression) to using are exposed to high availability [51]. Based on that premise, areas with both high availability of non-prescription opioids (metropolitan areas) and proneness could have higher overdose mortality rates, and consequently higher odds of being a mismatch county. The potentially synergistic association between unemployment rates and depression with high rates of opioid overdose mortality and access to MOUD should be further explored.

In non-metropolitan counties, a higher rate of uninsured individuals was associated with higher odds of a county being mismatch, but a significant association was not observed in metropolitan counties. Uninsured adults with OUD are particularly vulnerable because of limited access to treatment and care [52]. While overdose rates are higher in metropolitan versus non-metropolitan counties, the association we observed between insurance rates and a county being mismatch in non-metropolitan counties may be driven by lack of access to mental health and substance use services, a problem that is much more prominent in non-metropolitan counties [52]. Additionally, the higher rates of disability and chronic conditions as well as mental illness in uninsured populations may further hinder access to necessary healthcare services [52].

Although we did not stratify our analysis based on opioid type, our findings are consistent with national data showing higher opioid poisoning in urban areas, specifically from heroin and synthetic opioids, whereas higher rates of deaths in rural areas from semisynthetic opioids are reported (e.g. oxycodone, hydrocodone, and codeine) [53]. The more urban-centered opioid overdose epidemic from 2016 to 2020 [4,5] could have categorized metropolitan areas as mismatch despite the higher availability of resources in metropolitan areas. While rurality is a known persistent risk factor to treatment access [54–56], the characteristics of mismatch counties that arose from our results indicate that more resources should be targeted to metropolitan areas. It is also possible that our definition of risk-availability mismatch was not sufficiently granular and that some counties that were not categorized as mismatch were in reality mismatch counties because the available facilities did not provide the full portfolio of options for MOUD care. Some counties could have been classified as non-mismatch despite having high overdose rates because their facility rate per 100,000 population was above the national average, but we do not know if these facilities only provided naltrexone, which is known to be less effective than buprenorphine and methadone [57]. However, we found that nearly 88% of the facilities in our sample that prescribe MOUD provided at least methadone or buprenorphine. Additionally, among facilities that prescribe MOUD, the proportion of naltrexone-only facilities was comparable in mismatch and non-mismatch counties (S4 Table).

Our results are congruent with previous research that examined state- and county-level ratios between treatment programs and mortality rates called programs-per-death (PPD) [58]. Langabeer et al [58] showed high PPD rates primarily in the north central part of the US, including Wyoming, North Dakota, South Dakota, Montana, Nebraska, and Vermont. This is largely driven by the low overdose rates and disproportionately more programs per 100,000 population in these areas. Risk-availability mismatch counties represent counties where the demand for resources is highest. Improving resources can include increasing numbers of primary care physicians and other clinicians willing to provide MOUD, or other innovative strategies such as telemedicine, access to non-physician prescribers such as pharmacists, addressing stigma, closing the coverage gap, and expanding Medicaid services among unemployed and low-income individuals [59–62]. Without expansion of resources, there will continue to be long wait times for patients seeking treatment, with studies showing that it sometimes takes weeks or months to get individuals into treatment [63,64].

In line with other studies showing that many of the counties that lacked opioid treatment facilities also lacked office-based opioid treatment (where MOUDs are most likely to be prescribed despite changes following X-waiver) [28,56], we observed a strong correlation (row=0.88) between the availability of facilities and the availability of buprenorphine providers. We found that 83.2% of counties with below-average facility availability are also below-average in terms of buprenorphine provider availability.

## Limitations

Our study is not without limitations. One of the limitations of the analysis is the use of a general definition for OUD treatment availability. OUD treatment availability is more complex than merely the presence of opioid treatment facilities, and includes consideration of patient preferences, stigma, structural racism, and other access issues [17]. Second, our analysis was limited to data available through the CDC and PolicyMap which may not reflect the most recent landscape of care. Since 2019, there have been notable efforts to expand access to OUD treatment, especially in the context of the COVID-19 pandemic, a time where many changes were occurring in both the clinical and policy space (e.g. prescribing buprenorphine via telehealth without an initial in-person exam, take-home doses of methadone to relatively stable patients) [65]. However, we believe our study provides an important overview of the state of care and access needs that predates the COVID-19 pandemic. Third, the information on SAMHSA Behavioral Health Treatment Services Locator (findtreatment.gov) regarding services provided may have been inaccurate or there might have been changes to the kind of services offered at the facility (e.g. MOUDs) that were not updated on the website. Furthermore, the list of OUD treatment facilities includes any facility that provided or allowed MOUD, and therefore, we are not measuring providers specifically, but access to treatment programs. Each program is different in terms of their approach in OUD treatment and MOUD portfolio. Certain facilities that provide MOUD might only provide naltrexone (antagonist therapy), others provide behavioral therapy without prescribing MOUD even if they allow for patients to be on MOUD, while others might provide all 3 approved MOUDs and behavioral therapy. The scope of each facility and other features such as client volume capacity was not investigated directly, which is an inherent limitation of the data utilized. Fourth, accurately mapping geographic boundaries of care utilization is challenging as some individuals seek treatment across boundary lines. This may overestimate the challenges to accessing treatment facilities in smaller counties, but may underestimate the challenges in larger counties and clusters of contiguous counties that collectively have low rates of facilities. Finally, associations described in the study are ecologic and cannot be generalized to individuals within counties.

## Conclusion

Our study adds more information on the burden of opioid overdose at the population level for counties in the US. Assessing the relationship between availability (facilities) and need (overdose deaths) provides useful information to better identify geographic areas where there is a significant mismatch. Our results illuminate the multitude of characteristics that define counties where resources should be targeted to have the greatest potential of increasing treatment and reducing overdose mortality. Interventions are unlikely to be effective if they do not consider the diverse sociodemographic (e.g. unemployment, insurance status), disease burden (e.g. depression), and geographic context of communities, which could be upstream contributors to the opioid crisis.

## Supporting information

S1 Fig The geographic distribution of risk-availability mismatch counties, where mismatch is defined as counties that have below national rates of opioid treatment facilities *and* buprenorphine providers in 2019, and above national rates of overdose mortality in 2018. (PNG)

S2 Fig The geographic distribution of risk-availability mismatch counties, where mismatch is defined as counties that have below national rates of buprenorphine providers in 2019, and above national rates of overdose mortality in 2018. (PNG)

S3 Fig The geographic distribution of risk-availability mismatch counties, mid-group counties, and non-mismatch counties. Mid-group counties are those with drug overdose mortality rates that are within 0.5 standard deviations of the national average for overdose mortality deaths, and OUD treatment facility rates that are within 0.5 standard deviations of the national average of OUD treatment facilities.
(PNG)

S1 Table United States county-level characteristics associated with a mismatch between opioid overdose mortality and availability of opioid treatment facilities, univariate analyses (n=3,130). *P<0.05, **P<0.01, ***P<0.001. Model was adjusted for all variables above. To account for nesting of counties in states, a random effect model was used.
(DOCX)

S2 Table United States county-level characteristics associated with a mismatch between opioid overdose mortality and availability of opioid treatment facilities and/or buprenorphine providers, multivariable analysis (n=3,130). a. Counties with high overdose rates and below average rates of buprenorphine provider availability and facility availability b. Counties with high overdose rates and below average rates of buprenorphine provider availability. *P<0.05, **P<0.01, ***P<0.001. Model was adjusted for all variables above. To account for nesting of counties in states, a random effect model was used.
(DOCX)

S3 Table United States county-level characteristics associated with a mismatch between opioid overdose mortality and availability of opioid treatment facilities (versus non-mismatch counties), accounting for mid-group counties^a^, multivariable analysis (n=3,130). a. Mid-group counties are those with drug overdose mortality rates that are within 0.5 standard deviations of the national average for overdose mortality deaths, and OUD treatment facility rates that are within 0.5 standard deviations of the national average of OUD treatment facilities. *P<0.05, **P<0.01, ***P<0.001. Model was adjusted for all variables above. To account for nesting of counties in states, a random effect model was used.
(DOCX)

S4 Table Characteristics of facilities in risk-availability mismatch versus non-mismatch counties. a. Row percentages. b. Column percentages. c. Facilities that prescribe 1 or more of the following: buprenorphine, methadone, and/or naltrexone.
(DOCX)

S1 File (PDF)
