# Supplementary figures and images for: County-level factors associated with a mismatch between opioid overdose mortality and availability of opioid treatment facilities
Source: PLoS One. 2024 Apr 5;19(4):e0301863. doi: 10.1371/journal.pone.0301863 (PMC10997118; doi:10.1371/journal.pone.0301863)

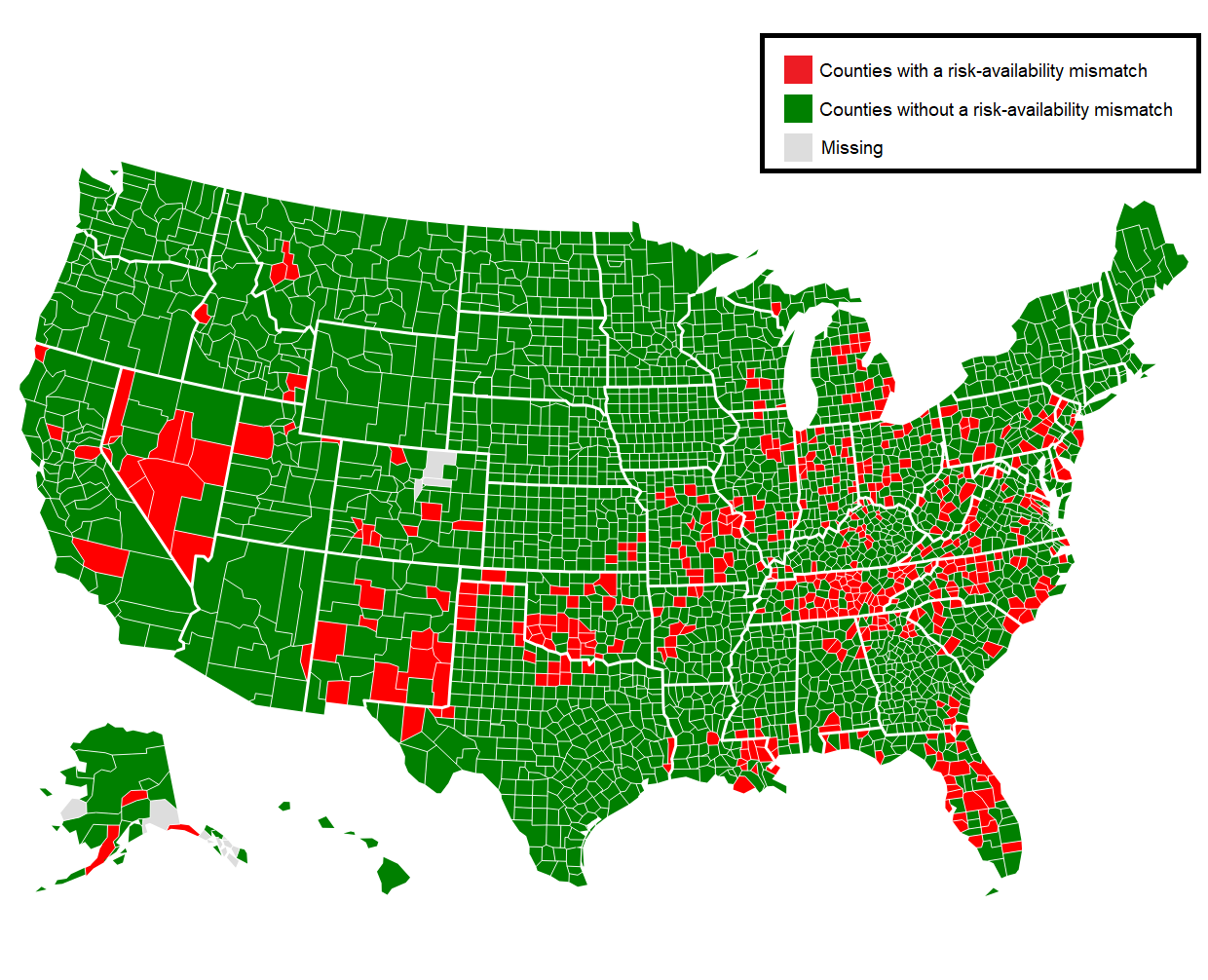

Supplement: S1 Fig — (PNG) [file pone.0301863.s001.png]

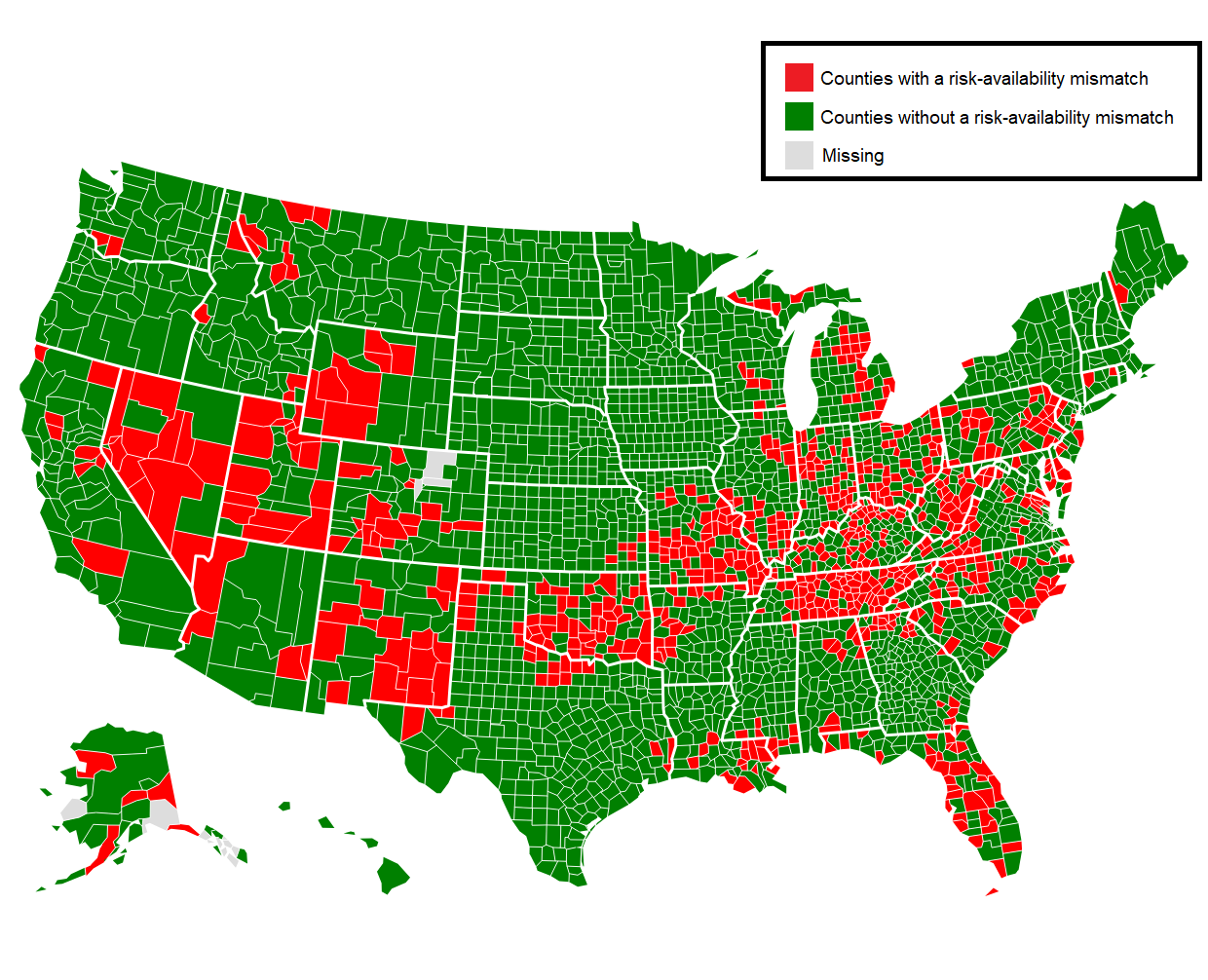

Supplement: S2 Fig — (PNG) [file pone.0301863.s002.png]

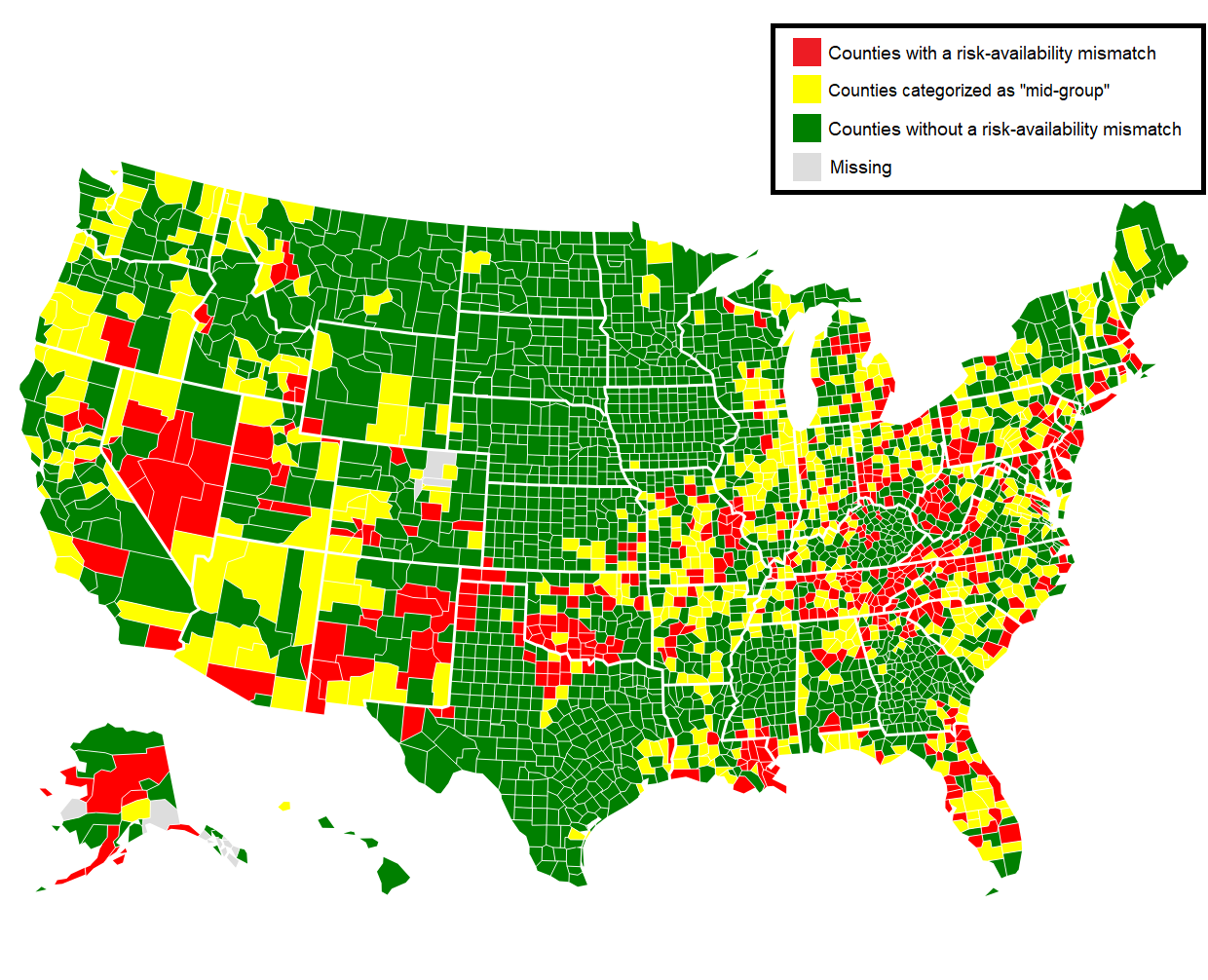

Supplement: S3 Fig — Mid-group counties are those with drug overdose mortality rates that are within 0.5 standard deviations of the national average for overdose mortality deaths, and OUD treatment facility rates that are within 0.5 standard deviations of the national average of OUD treatment facilities. (PNG) [file pone.0301863.s003.png]
