# Supplementary material for: County-level factors associated with a mismatch between opioid overdose mortality and availability of opioid treatment facilities
Source: PLoS One. 2024 Apr 5;19(4):e0301863. doi: 10.1371/journal.pone.0301863 (PMC10997118; doi:10.1371/journal.pone.0301863)
Supplement: S1 Table — *P<0.05, **P<0.01, ***P<0.001. Model was adjusted for all variables above. To account for nesting of counties in states, a random effect model was used. (DOCX) [file pone.0301863.s004.docx]

**S1 Table.** **United States county-level characteristics associated with a mismatch between opioid overdose mortality and availability of opioid treatment facilities, univariate analyses (n=3,130).** *P<0.05, **P<0.01, ***P<0.001. Model was adjusted for all variables above. To account for nesting of counties in states, a random effect model was used.

| **Characteristics** | **Crude Odds Ratio (95% CI)** |
| --- | --- |
| Rate of opioid prescriptions per 100 population | 1.00 (1.00-1.00) |
| Sex, % male | 0.98 (0.94-1.02) |
| Race, % white | 1.02 (1.01-1.03)*** |
| % Age 18-64 y | 1.02 (0.99-1.04) |
| Theil Index |  |
| <0.4 | **1 (reference)** |
| ≥0.4 | 0.87 (0.71-1.07) |
| Urbanicity |  |
| Non-metropolitan | **1 (reference)** |
| Metropolitan | 1.58 (1.29-1.92)*** |
| **Socioeconomic factors** | |
| % Unemployment | 1.03 (0.97 -1.08) |
| % Less than high school degree | 0.99 (0.97-1.01) |
| % Poverty | 0.97 (0.95-0.99)*** |
| % Uninsured | 1.00 (0.97-1.03) |
| Gini Index |  |
| <0.45 | **1 (reference)** |
| ≥0.45 | 0.83 (0.68-1.00) |
| **Clinical factors** | |
| % Heart disease | 1.00 (0.94-1.07) |
| % Depression | 1.08 (1.02-1.15)* |

*P<0.05, **P<0.01, ***P<0.001. Model was adjusted for all variables above. To account for nesting of counties in states, a random effect model was used.
