# Supplementary material for: County-level factors associated with a mismatch between opioid overdose mortality and availability of opioid treatment facilities
Source: PLoS One. 2024 Apr 5;19(4):e0301863. doi: 10.1371/journal.pone.0301863 (PMC10997118; doi:10.1371/journal.pone.0301863)
Supplement: S2 Table — a. Counties with high overdose rates and below average rates of buprenorphine provider availability and facility availability b. Counties with high overdose rates and below average rates of buprenorphine provider availability. *P<0.05, **P<0.01, ***P<0.001. Model was adjusted for all variables above. To account for nesting of counties in states, a random effect model was used. (DOCX) [file pone.0301863.s005.docx]

**S2 Table. United States county-level characteristics associated with a mismatch between opioid overdose mortality and availability of opioid treatment facilities and/or buprenorphine providers, multivariable analysis (n=3,130).**

| **Characteristics** | **Adjusted Odds Ratio (95% CI)** | |
| --- | --- | --- |
|  | **Mismatch based on opioid treatment facilities and buprenorphine provider^a^** | **Mismatch based on buprenorphine providers only^b^** |
| Rate of opioid prescriptions per 100 population | 1.00 (0.99-1.00) | 1.00 (0.99-1.00) |
| Sex, % male | 0.99 (0.93-1.05) | 1.05 (0.99-1.11) |
| Race, % white | 1.03 (1.01-1.04)*** | 1.03 (1.02-1.04)*** |
| % Age 18-64 y | 1.00 (0.95-1.07) | 1.05 (1.00-1.11) |
| Theil Index |  |  |
| <0.4 | **1 (reference)** | **1 (reference)** |
| ≥0.4 | 0.86 (0.65-1.13) | 0.84 (0.66-1.07) |
| Urbanicity |  |  |
| Non-metropolitan | **1 (reference)** | **1 (reference)** |
| Metropolitan | 1.59 (1.20-2.10)** | 1.32 (1.02-1.70)* |
| **Socioeconomic factors** | |  |
| % Unemployment | 1.10 (1.02-1.18)* | 1.17 (1.10-1.25)*** |
| % Less than high school degree | 1.04 (1.00-1.08)* | 1.01 (0.98-1.05) |
| % Poverty | 0.96 (0.92-1.00)* | 0.92 (0.89-0.96)*** |
| % Uninsured | 1.02 (0.98-1.06) | 1.04 (1.00-1.08)* |
| Gini Index |  |  |
| <0.45 | **1 (reference)** | **1 (reference)** |
| ≥0.45 | 0.70 (0.54-0.91)** | 0.76 (0.61-0.96)* |
| **Clinical factors** | |  |
| % Heart disease | 1.09 (0.91-1.29) | 1.48 (1.27-1.73)*** |
| % Depression | 1.07(0.96-1.20) | 1.20 (1.09-1.34)*** |

a. Counties with high overdose rates and below average buprenorphine provider availability and facility availability b. Counties with high overdose rates and below average buprenorphine provider availability. *P<0.05, **P<0.01, ***P<0.001. Model was adjusted for all variables above. To account for nesting of counties in states, a random effect model was used.
