# Supplementary material for: County-level factors associated with a mismatch between opioid overdose mortality and availability of opioid treatment facilities
Source: PLoS One. 2024 Apr 5;19(4):e0301863. doi: 10.1371/journal.pone.0301863 (PMC10997118; doi:10.1371/journal.pone.0301863)
Supplement: S3 Table — a. Mid-group counties are those with drug overdose mortality rates that are within 0.5 standard deviations of the national average for overdose mortality deaths, and OUD treatment facility rates that are within 0.5 standard deviations of the national average of OUD treatment facilities. *P<0.05, **P<0.01, ***P<0.001. Model was adjusted for all variables above. To account for nesting of counties in states, a random effect model was used. (DOCX) [file pone.0301863.s006.docx]

**S3 Table. United States county-level characteristics associated with a mismatch between opioid overdose mortality and availability of opioid treatment facilities (versus non-mismatch counties^a^), accounting for mid-group counties^b^, multivariable analysis (n=3,130).**

| **Characteristics** | **Adjusted Odds Ratio (95% CI)** | | |
| --- | --- | --- | --- |
|  | **Mid-group (n=632)** | | **Mismatch (n=525)** |
| Rate of opioid prescriptions per 100 population | 1.00 (1.00-1.00) | | 1.00 (1.00-1.00) |
| Sex, % male | 0.97 (0.91-1.03) | | 0.96 (0.90-1.02) |
| Race, % white | 1.01 (0.99-1.02) | | 1.02 (1.01-1.03)*** |
| % Age 18-64 y | 0.99 (0.92-0.99) | | 1.04 (0.98-1.10) |
| Theil Index |  | |  |
| <0.4 | **1 (reference)** | | **1 (reference)** |
| ≥0.4 | 0.67 (0.51-0.87) | | 1.04 (0.79-1.37) |
| Urbanicity |  | |  |
| Non-metropolitan | **1 (reference)** | | **1 (reference)** |
| Metropolitan | 1.18 (0.91-1.52) | | 1.91 (1.43-2.54)*** |
|  | | **Socioeconomic factors** | |
| % Unemployment | 1.20 (1.12-1.28)*** | | 1.10 (1.02-1.18)* |
| % Less than high school degree | 0.97 (0.94-1.01) | | 1.01 (0.98-1.05) |
| % Poverty | 0.96 (0.92-0.99)* | | 0.96 (0.92-0.99)* |
| % Uninsured | 1.03 (1.00-1.07) | | 1.05 (1.01-1.09)* |
| Gini Index |  | |  |
| <0.45 | **1 (reference)** | | **1 (reference)** |
| ≥0.45 | 1.18 (0.93-1.50) | | 0.87 (0.68-1.13) |
|  | | **Clinical factors** | |
| % Heart disease | 0.83 (0.70-0.98)* | | 1.15 (0.98-1.36) |
| % Depression | 1.19 (1.09-1.30)*** | | 1.04 (0.94-1.15) |

a. n=1,973. b. Mid-group counties are those with drug overdose mortality rates that are within 0.5 standard deviations of the national average for overdose mortality deaths, and OUD treatment facility rates that are within 0.5 standard deviations of the national average of OUD treatment facilities.

*P<0.05, **P<0.01, ***P<0.001. Model was adjusted for all variables above. To account for nesting of counties in states, a random effect model was used.
