# Supplementary material for: County-level factors associated with a mismatch between opioid overdose mortality and availability of opioid treatment facilities
Source: PLoS One. 2024 Apr 5;19(4):e0301863. doi: 10.1371/journal.pone.0301863 (PMC10997118; doi:10.1371/journal.pone.0301863)
Supplement: S4 Table — a. Row percentages. b. Column percentages. c. Facilities that prescribe 1 or more of the following: buprenorphine, methadone, and/or naltrexone. (DOCX) [file pone.0301863.s007.docx]

**S4 Table. Characteristics of facilities in risk-availability mismatch versus non-mismatch counties (n=3,130).**

| **Characteristics** | **All**  **counties** | **Non-mismatch Counties** | **Mismatch Counties** |
| --- | --- | --- | --- |
| Total number of facilities, n (%)^a^ | 11,111 | 8,054 (72.49%) | 3,057 (27.51%) |
| Allows medications but must be provided by outside prescriber or facility, n (%)^b^ | 4,929 (44.36%) | 3,761 (46.70%) | 1,168 (38.21%) |
| Medications provided on-site, n (%)^b,c^ | 6,182 (55.64%) | 4,293 (53.30%) | 1,889 (61.79%) |
| Provided at least buprenorphine or methadone, n (%)^b^ | 5,445 / 6,182 (88.08%) | 3786 / 4,293 (88.19%) | 1,659 / 1,889 (87.82%) |
| Provided naltrexone only, n (%)^b^ | 737 / 6,182 (11.92%) | 507 / 4,293 (11.81%) | 230 / 1,889 (12.18%) |

1. Row percentages.
2. Column percentages.
3. Facilities that prescribe 1 or more of the following: buprenorphine, methadone, and/or naltrexone.
